# Beneficial effect of medial opening wedge high tibial osteotomy on mental health of patients with knee osteoarthritis

**DOI:** 10.1002/jeo2.70400

**Published:** 2025-09-04

**Authors:** Ashton Kai Shun Tan, Xinyu Tao, Shao Jin Teo, Don Thong Siang Koh, Junwei Soong, Hamid Rahmatullah Bin Abd Razak, Kong Hwee Lee

**Affiliations:** ^1^ Department of Orthopaedic Surgery Singapore General Hospital Singapore Singapore; ^2^ Total Orthopaedic Care & Surgery Singapore Singapore

**Keywords:** high tibial osteotomy, knee preservation, mental health, osteoarthritis

## Abstract

**Purpose:**

Knee osteoarthritis (OA) is the most common degenerative joint disease in the world. It results in not only physical limitation and pain, but also negatively impacts quality of life and mental wellbeing. Treatment options such as medial opening wedge high tibial osteotomy (MOWHTO) help to relieve pain and improve functionality, but there is limited literature on the effect on mental health. This study aims to investigate the effect of MOWHTO on the mental health of patients with knee OA.

**Methods:**

In this retrospective cohort study, data were collected from patients who underwent MOWHTO between 2019 and 2023 in a single tertiary institution. The main outcome score, short form‐36 mental component score (SF‐36 MCS), was recorded at baseline, 6 months and 2 years postprocedure. Secondary outcomes included the SF‐36 physical component score (PCS) and Oxford knee score (OKS).

**Results:**

There were a total of 108 MOWHTOs, comprising 51 men and 57 women. At surgery, the mean age was 55.4 ± 6.97. Twelve patients did not turn up at the 6‐month follow‐up. There was a significant improvement of SF‐36 MCS from a preoperative mean of 50.8 ± 12.6 to 57.1 ± 11.0 (*p* < 0.05) and 56.8 + 9.07 (*p* < 0.05) at 6 months and 2 years postoperation, respectively. There was also a significant increase in the scores of SF‐36 PCS and OKS (*p* < 0.05 for both) at 6‐month and 2‐year follow‐up. Higher preoperative SF‐36 MCS was significantly correlated with improved postoperation SF‐36 PCS (*ρ* = 0.184, *p* = 0.05) and OKS (*ρ* = −0.218, *p* = 0.02 < 0.05).

**Conclusions:**

At 2‐year follow‐up, MOWHTO results in a significant improvement in the mental health of patients with knee OA. There is a correlation between preoperative mental health and postoperative outcomes, suggesting a role of mental health in affecting MOWHTO outcomes.

**Level of Evidence:**

Level IV.

**Abbreviations**
AKO: arthroplasty and around knee osteotomy
BMI: body mass index
MOWHTO: medial opening wedge high tibial osteotomy
OA: osteoarthritis
OKS: Oxford knee score
SF‐36 MCS: short form‐36 mental component score
SF‐36 PCS: short form‐36 physical component score
TKA: total knee arthroplasty
UKA: unicompartmental knee arthroplasty

## INTRODUCTION

Knee osteoarthritis (OA) is one of the most common degenerative joint diseases in the world, and a leading cause of disability [7]. Patients face symptoms and complications such as pain, reduction in mobility as well as function [6]. While the physical impact of knee OA has been well described, the impact on mental well‐being is now increasingly recognised. Park et al. found a close relationship between knee OA and poor mental health, and attributed this to poor sleep due to pain and altered serotonin transmission due to increased pro‐inflammatory cascades [16]. Da silva et al. suggested that disability and reliance resulting from pain could lead to a vicious cycle, where patients are socially isolated or institutionalised and develop susceptibility to psychological disorders [18].

The mainstay of surgical treatment of knee OA includes arthroplasty and around knee osteotomy (AKO) [6, 9]. Good and sustained clinical outcomes have been reported with both options [4, 9]. Several studies have also suggested that total knee arthroplasty (TKA) and unicompartmental knee arthroplasty (UKA) improve mental health. Horst et al. found that TKA was effective in improving the mental health of patients, including those with low initial baseline scores [8]. Plancher et al. reported that UKA resulted in an improvement in mental health postoperatively [17]. However, there is currently limited literature on the effect of AKO on mental health.

To fill this gap in literature, this study aims to evaluate and report on the mental wellbeing of a patient cohort undergoing Medial Opening Wedge High Tibial Osteotomy (MOWHTO) for the treatment of knee OA. The authors hypothesise that patients may experience significant improvement in mental wellbeing alongside physical wellbeing after undergoing MOWHTO.

## METHODS

### Study design

This was a retrospective cohort study of prospectively collected data conducted at a single tertiary centre.

### Patient selection

All patients with knee OA who underwent MOWHTO between January 2019 to January 2023 were identified from the institutional database. All cases were performed by a single fellowship‐trained senior knee surgeon in our tertiary institution. Participants were included regardless of pre‐existing mental health or psychiatric conditions, if they were above the age of 18 and underwent elective MOWHTO for medial compartmental knee OA. Patients who had bilateral procedures, concomitant ligamentous procedure, previous knee surgeries, incomplete medical records or incomplete follow‐up were excluded from this study.

### Surgical procedure

All patients underwent MOWHTO with an ascending biplanar cut. This was to improve rotational stability and increase surface area for bony union. Femoral head allograft was used to fill the osteotomy wedge. The osteotomy was stabilised with a locking plate (Activmotion S, Newclip Technics). Preoperative planning was performed with the aid of a digital templating software program (TraumaCad®, Voyant Health, BrainLab). The opening wedge dimensions are determined by the correction required to shift the mechanical axis to the apex of the lateral tibial spine.

### Postoperative rehabilitation

In the absence of intraoperative complications, patients were allowed full weightbearing and range of movement as tolerated. They were started on rehabilitation with the physiotherapists from postoperative Day 1 and discharged once assessed to be fit for home. Analgesia was prescribed with accordance to the World Health Organisation (WHO) pain ladder, with a general regime of paracetamol and etoricoxib with omeprazole cover. If the patient was unable to tolerate etoricoxib, tramadol was prescribed instead.

### Data collection

Patient demographics including age, gender, body mass index (BMI) and previous psychiatric history were extracted from electronic medical records. Clinical outcome scores were collected preoperatively, at 6 months and 2 years after MOWHTO. The main outcome was the short form‐36 mental component score (SF‐36 MCS), and secondary outcomes included the SF‐36 physical component score (PCS) and Oxford knee score (OKS). These were collected by a team of experienced physiotherapists as part of routine departmental protocol.

The SF‐36 evaluates health‐related quality of life [12]. It comprises eight domains: physical functioning (PF), role physical (RP), bodily pain (BP), general health (GH), vitality (VT), social functioning (SF), role emotional (RE) and mental health (MH). The SF‐36 physical component score (PCS) and mental component score (MCS) are derived from these domains [11]. SF‐36 MCS of 50 represents the mean general population score with a standard deviation of 10 [10, 11]. Further subgroup analysis was performed, where patients with MCS of less than 50 were operationally defined as the lower mental health (LMH) group and the rest as the normal mental health (NMH) group.

The OKS is a patient‐reported measure of knee pain and function consisting of 12 items [5, 15]. It was initially devised to evaluate outcomes post‐TKA, but has since been modified for use for different purposes. A lower score reflects a better outcome.

### Statistical analysis

Baseline patient characteristics were reported descriptively. Data analysis was performed using Microsoft Excel Version 16.92 with XLSTAT and R (Version 2024.09.0 + 375, tidyverse and stats packages). Continuous variables were expressed as means with standard deviations and compared using either independent *t*‐tests or Mann–Whitney *U* tests depending on normality. Categorical variables were presented as frequencies and percentages, then analysed using chi‐squared tests.

The Pearson correlation coefficient was used to analyse the correlational relationship between preoperative SF‐36 MCS scores and postoperative outcomes (PCS and OKS). The level of statistical significance was set at ≤0.05.

## RESULTS

**Figure 1:**
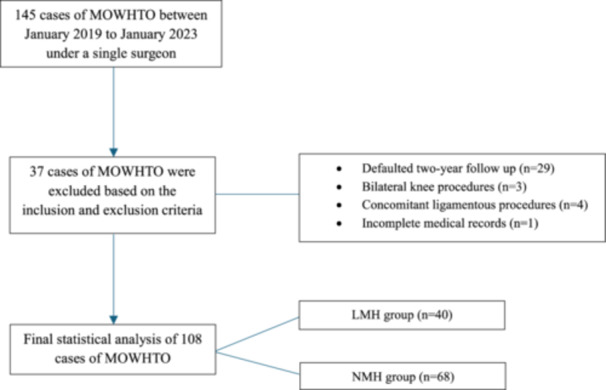
CONSORT flow diagram.

After 37 patients were excluded, a total of 108 cases were included for statistical analysis (Figure 1). The average age was 55.4 ± 6.97 with a mean BMI of 28.8 ± 5.14. 51% of included participants were males. None of the patients had pre‐existing mental health or psychiatric conditions. Further patient characteristics are summarised in Table 1. While 10 patients defaulted on the 6‐month follow‐up, all included patients attended the 2‐year follow‐up.

**Table 1:** Patient demographics.

Number of participants (*n*)	108
Age (mean ± SD)/(range)	55.4 ± 6.97/45–69
Male (%)	51 (47.2)
Body mass index (mean ± SD)	28.8 ± 5.14
KL^a^ grade (2/3/4)	23/36/49

a Kellgren–Lawrence (KL) classification for osteoarthritis.

Table 2 summarises the clinical scores collected preoperatively, at 6 months and 2 years after surgery. There was a significant improvement of SF‐36 MCS from a preoperative mean of 50.8 ± 12.6 to 57.1 ± 11.0 (*p* < 0.05) and 56.8 ± 9.07 (*p* < 0.05) at 6 months and 2 years postoperation, respectively. There was also a significant improvement in the scores of SF‐36 PCS and OKS (*p* < 0.05 for both) at both 6‐month and 2‐year follow‐up. These changes were all above the MCID for each respective score.

**Table 2:** Summary of patient‐reported outcomes.

	Preop (*n* = 108)	6 months (*n* = 98)		2 years (*n* = 108)	
	Score	Score	*p* value*	Score	*p* value*
Short form‐36 physical component score	32.7 (7.76)	43.7 (10.0)	<0.001	47.6 (9.40)	<0.001
Short form‐36 mental component score	50.8 (12.6)	57.1 (11.0)	<0.001	56.8 (9.07)	< 0.001
Oxford knee score	35.5 (10.8)	22.2 (7.75)	<0.001	18.8 (6.31)	<0.001

* *p* value compared to preoperative scores.

There was a weak but significant correlation between higher preoperative SF‐36 MCS and improved postoperative SF‐36 PCS (*ρ* = 0.184, *p* = 0.05) and OKS (*ρ* = −0.218, *p* = 0.02) scores. On further subgroup analysis, 40 participants had preoperative SF‐36 MCS scores below 50 (LMH group), while the remaining 68 had MCS scores above 50 (NMH group). There was no significant difference in baseline characteristics between the two groups (Table 3). Both groups had significant improvement in postoperative SF‐36 PCS and OKS scores (Table 4). At 2 years, the NMH group had no significant change in mental health scores, while the LMH group had significant improvement in SF‐36 MCS. On comparison of scores between the two groups, the NMH group had significantly higher MCS, PCS and OKS scores preoperatively. This result remained persistent at 2‐year follow‐up.

**Table 3:** Baseline characteristics of patients in lower mental health (LMH) and normal mental health (NMH) groups.

	LMH group (*n* = 40)	NMH group (*n* = 68)	*p* value
Age	54.5 ± 6.37	56.0 ± 7.28	0.295
Male (%)	15 (37.5)	36 (52.9)	0.120
Body mass index (mean ± SD)	30.1 ± 6.06	28.2 ± 4.53	0.0552
Kellgren–Lawrence grade 2/3/4	8/14/18	15/22/31	0.200

**Table 4:** Summary of patient‐reported outcomes compared between lower mental health (LMH) and normal mental health (NMH) groups.

	LMH group (*n* = 40)	NMH group (*n* = 68)	*p* value*
Short form‐36 mental component score	Preop: 37.0 ± 8.74 Postop: 54.2 ± 9.90 *p*‐Value**: <0.001	Preop: 58.8 ± 5.50 Postop: 58.3 ± 8.27 *p*‐Value**: <0.310	<0.001 0.0248
Short form‐36 physical component score	Preop: 29.8 ± 6.12 Postop: 45.11 ± 10.1 *p*‐Value**: <0.001	Preop: 34.5 ± 8.14 Postop: 49.04 ± 8.75 *p*‐Value**: <0.001	0.00225 0.0354
Oxford knee score	Preop: 41.7 ± 7.72 Postop: 20.6 ± 7.73 *p*‐Value**: <0.001	Preop: 31.8 ± 10.7 Postop: 17.7 ± 5.08 *p*‐Value**: <0.001	<0.001 0.0220

* *p* value comparing scores between the two groups

** *p* value comparing preoperative (preop) and postoperative scores (postop) (final follow‐up) within each group.

## DISCUSSION

This study found a beneficial impact on mental health after HTO for knee OA, especially in patients with impaired mental health preoperatively. This is of great clinical significance, as it adds to evidence that the benefits of surgical treatment of knee OA extend beyond the physical and functional domains.

While the functional and physical outcomes of surgical treatment for knee OA (both arthroplasty and AKOs) have long been shown, there has been less focus on their effect on patients' mental wellbeing [2, 4]. However, this has changed in recent years, as the importance of mental health gains increasing recognition. There is now growing awareness amongst clinicians that the disability from knee OA extends to the mental domain. Del val et al. reports a high incidence of depression and anxiety associated with knee OA [14]. Recent studies have found promising results for TKA and UKA [8]. At a mean of 10 years follow‐up, Plancher et al. reported that individuals with normal mental health preoperatively were more likely to achieve a patient acceptable symptom state for both pain and function after UKA [17]. However, no study has reported on the impact of HTO on mental health or looked into the relationship between mental health and outcomes post‐HTO.

HTO works by correcting the alignment of the lower limb to redistribute pressure across the joint line [1]. This optimises biomechanics and functional outcomes, preserves the native knee and reduces pain [1]. We postulate that better mobility and reduction of previous activity limitations will improve quality of life. Pain is also known to have a strong relationship with mental health [20]. A reduction of pain and better quality of life will eventually lead to better overall mental health. However, for individuals starting off with better mental health, although still present, these effects may not be as significant due to the ceiling effect [3]. This has been reported in the context of TKA, where Horst et al. found that those with higher baseline mental health did not experience as much improvement in postoperative mental health [8]. Similarly, this was also observed in our study where the NMH group did not experience a significant improvement of MCS as compared to the LMH group.

While we report in this study that HTO can beneficially impact mental well‐being, the converse is also true. Patient's mental well‐being can be an important factor in determining surgical outcomes as well. This has been well described in the context of knee arthroplasty. Scott et al. reported a relationship between low preoperative mental health and poorer outcomes after TKA [19]. Melnic et al. found that despite all patients experiencing similar improvement in the first year despite initial mental health, those who had poorer mental health scores experienced a deterioration in physical function after a year of TKA [13]. We hypothesise that more mentally positive patients would have a greater motivation to participate in postoperative rehabilitation and have a higher drive to recover and return to their initial baseline activity. There could also be a component of the pain and mental health cycle, where those with poorer mental health had a lower pain threshold, which in turn further affects their mental health—forming a vicious cycle. By comparing between LMH and NMH groups, it was observed that poorer preoperative MCS was associated with significantly lower preoperative PCS and OKS scores. This was despite a similar distribution of radiographic OA severity and patient biodemographic. This disparity was persistent at 2‐year follow‐up. Our findings suggest that there is a role of an individual's mental health in influencing perceived physical outcomes. However, the authors recognise that the design of this study does not allow us to definitively rule out the possibility that lower MCS could be a result of more severe OA knees to begin with. Moreover, the authors acknowledge that the correlation observed in our study is weak (although significant). Future studies with larger cohorts and longer follow‐up are crucial to further investigate this.

The relationship between mental health and postoperative outcomes is crucial, as this affects clinicians' decision‐makingand counselling process when offering surgical options. We suggest that clinicians evaluate or minimally screen their patients' mental health prior to arranging for operation. For those with lower mental health who are still keen for surgery, it may be beneficial to first optimise their mental health. This could include a referral to a psychologist or therapist, medication for those with a proper psychiatric diagnosis, or joining a focus group for support. With more data and studies, it would be useful to eventually create an algorithm to guide clinicians—from concurrent screening of mental health during MOWHTO planning, to mental health score cut offs that determine the need for intervention and optimal time that surgery can be done.

The authors recognise several limitations in this study. First, the population size was small and only restricted to an Asian population in our institution. Second, none of our included patients had pre‐existing mental health or psychiatric conditions, thus this group may be underrepresented. Next, follow‐up was relatively short at 2 years. Lastly, more sensitive mental health scales could have been utilised to obtain a more holistic mental health assessment of participants.

Future studies could focus on long term effects of HTO on mental health, with a larger population size. Since we noted a mild and insignificant decrease in MCS at 2 years, it would be interesting to observe whether mental health scores are maintained in the long run. It would be interesting to investigate the extent of mental health improvement amongst TKA, UKA and HTO.

## CONCLUSION

At 2‐year follow‐up, MOWHTO results in a significant improvement in the mental health of patients with knee OA. There is a correlation between preoperative mental health and postoperative outcomes, suggesting a role of mental health in affecting MOWHTO outcomes.

## AUTHOR CONTRIBUTIONS

All authors contributed to the study conception and design. Data analysis and the first draft of the manuscript was written by Ashton Kai Shun Tan. All authors commented on previous versions of the manuscript. All authors read and approved the final manuscript.

## CONFLICT OF INTEREST STATEMENT

The authors declare no conflict of interest.

## ETHICS STATEMENT

Ethical approval was obtained from the institutional review board prior to data collection, and all procedures adhered to the principles outlined in the Declaration of Helsinki.

## Data Availability

The authors have nothing to report.
